# A Novel Conserved Protein in Streptococcus agalactiae, BvaP, Is Important for Vaginal Colonization and Biofilm Formation

**DOI:** 10.1128/msphere.00421-22

**Published:** 2022-10-11

**Authors:** Lamar S. Thomas, Laura C. Cook

**Affiliations:** a Binghamton Biofilm Research Center, Department of Biology, Binghamton University, Binghamton, New York, USA; University of Iowa

**Keywords:** adherence, colonization, streptococcus, vaginal

## Abstract

Streptococcus agalactiae (group B streptococcus [GBS]) infections in neonates are often fatal and strongly associated with maternal GBS vaginal colonization. Here, we investigated the role of an uncharacterized protein, BvaP, in GBS vaginal colonization. *bvaP* was previously identified as the most highly upregulated gene in the GBS A909 transcriptome when comparing vaginal colonization to growth in liquid culture. We found that the absence of BvaP affects the ability of GBS to adhere to extracellular matrix components and human vaginal epithelial cells, and the ability of a Δ*bvaP* mutant to colonize the murine vaginal tract was significantly decreased. Cellular morphological alterations such as changes in cell shape, chain length, and clumping were also observed in a knockout mutant strain. Given its high expression level *in vivo*, high degree of conservation among GBS strains, and role in vaginal colonization, BvaP may be an eligible target for GBS vaccination and/or drug therapy.

**IMPORTANCE** Neonatal GBS disease is a major cause of morbidity and mortality, and maternal vaginal colonization is the leading risk factor for the disease. Colonization prevention would greatly impact the rates of disease transmission, but vaccine development has stalled as capsular polysaccharide vaccines have low immunogenicity *in vivo.* While these vaccines are still in development, the addition of a protein conjugate may prove fruitful in increasing immunogenicity and strain coverage across GBS serotypes. Previous research identified *sak_1753* as a highly upregulated gene during murine vaginal colonization. This study reveals that Sak_1753 is required to maintain proper GBS cellular morphology and colonization phenotypes and is required for full *in vivo* vaginal colonization in a murine model. We have renamed Sak_1753 group B streptococcus vaginal adherence protein (BvaP). The findings of this study indicate that BvaP is important for GBS colonization of the vaginal tract and, given its high expression level *in vivo* and strain conservation, may be a candidate for vaccine development.

## INTRODUCTION

Streptococcus agalactiae (group B streptococcus [GBS]) colonizes the rectovaginal tract of approximately 10 to 35% of the general population. GBS is the leading cause of neonatal sepsis worldwide and accounts for many maternal and fetal bacterial infections, postinfection sequelae, and fetal death (1). GBS may also ascend into the uterus and cause *in utero* infections, which may result in premature birth and stillbirth. More commonly, GBS is transmitted vertically from a vaginally colonized gravid woman to her child during delivery, likely due to the aspiration of contaminated amniotic and bodily fluids, resulting in deadly invasive neonatal diseases. The use of preventive intrapartum antibiotics during labor in GBS-colonized women has been effective in lowering the rates of early-onset GBS disease but comes with pitfalls, including showing no large effect on late-onset neonatal GBS diseases (2) and alteration of the neonatal microbiota (3, 4).

The ability of GBS to colonize the vaginal mucosa is essential for the pathogenesis of neonatal diseases. While some colonization factors have been described for GBS, many are strain specific or not present in all GBS isolates obtained from colonized women or infected neonates. A more complete understanding of GBS vaginal colonization factors could open new avenues for the development of future therapeutics designed to prevent GBS vaginal carriage and neonatal infection without, or in addition to, intrapartum antibiotics.

Numerous studies have described examples of GBS interactions with extracellular matrix (ECM) components (5–8). The ECM of host mammalian tissue is composed of structural glycoproteins such as fibrinogen and fibronectin, which form a stable macromolecular structure surrounding endothelial and epithelial cells. Researchers have proposed that interactions between GBS and ECM components are important for bacterial invasion and host tissue adhesion. Not all described ECM-bacterium interactions are conserved in all strains of GBS, and as such, the set of surface proteins found in specific GBS strains influences their colonization and virulence potential. Currently, conserved surface proteins are being explored for vaccine development against pneumonia and sepsis caused by the related organism Streptococcus pneumoniae (9), and a similar strategy for GBS vaccination may prove fruitful. In this case, a highly conserved protein could prove useful as a new therapeutic target.

A recent study described the transcriptomic profile of GBS strain A909 growing in liquid culture and compared it to the profile of the same strain colonizing the murine vaginal tract for 48 h (10). A hypothetical gene, *sak_1753*, was identified as the most highly upregulated gene in the A909 transcriptome during vaginal colonization. A subsequent study used RNA sequencing (RNA-seq) to examine the transcriptome of GBS strain CJB111 colonizing the murine vaginal tract. This study also found that the *sak_1753* homolog (ID870_01035) was the most highly upregulated gene at day 3 of colonization and in the top 10 most highly upregulated genes at day 1 (11), with over 150-fold upregulation at both time points, showing conservation of this high *in vivo* expression level among different GBS strains.

A two-component system, SaeRS, was also significantly upregulated *in vivo* (10). In a strain deleted for the response regulator *saeR*, *sak_1753* as well as a known adhesin, *pbsP*, were no longer upregulated in the murine vaginal tract. Gel shift assays showed that a phosphomimetic mutant of SaeR directly regulates *pbsP* via promoter binding. P*_sak_1753_* has large regions identical to P*_pbsP_*, indicating the direct regulation of *sak_1753* by SaeR as well (10). Sak_1753 is present in every sequenced GBS strain that we have access to, although homologs outside GBS are confined to only a few species of streptococci and enterococci. Our data indicate that Sak_1753 influences GBS mucosal colonization by altering bacterial biofilm formation abilities, attachment to host ECM components, and colonization of the vaginal tract *in vivo*. The deletion of *sak_1753* also results in changes in the cell shape, chain length, and clumping ability. Based on this, we have named Sak_1753 group B streptococcus vaginal adherence protein (BvaP).

## RESULTS

### BvaP is a novel protein made up of repeated domains and is conserved among GBS strains.

**FIG 1:**
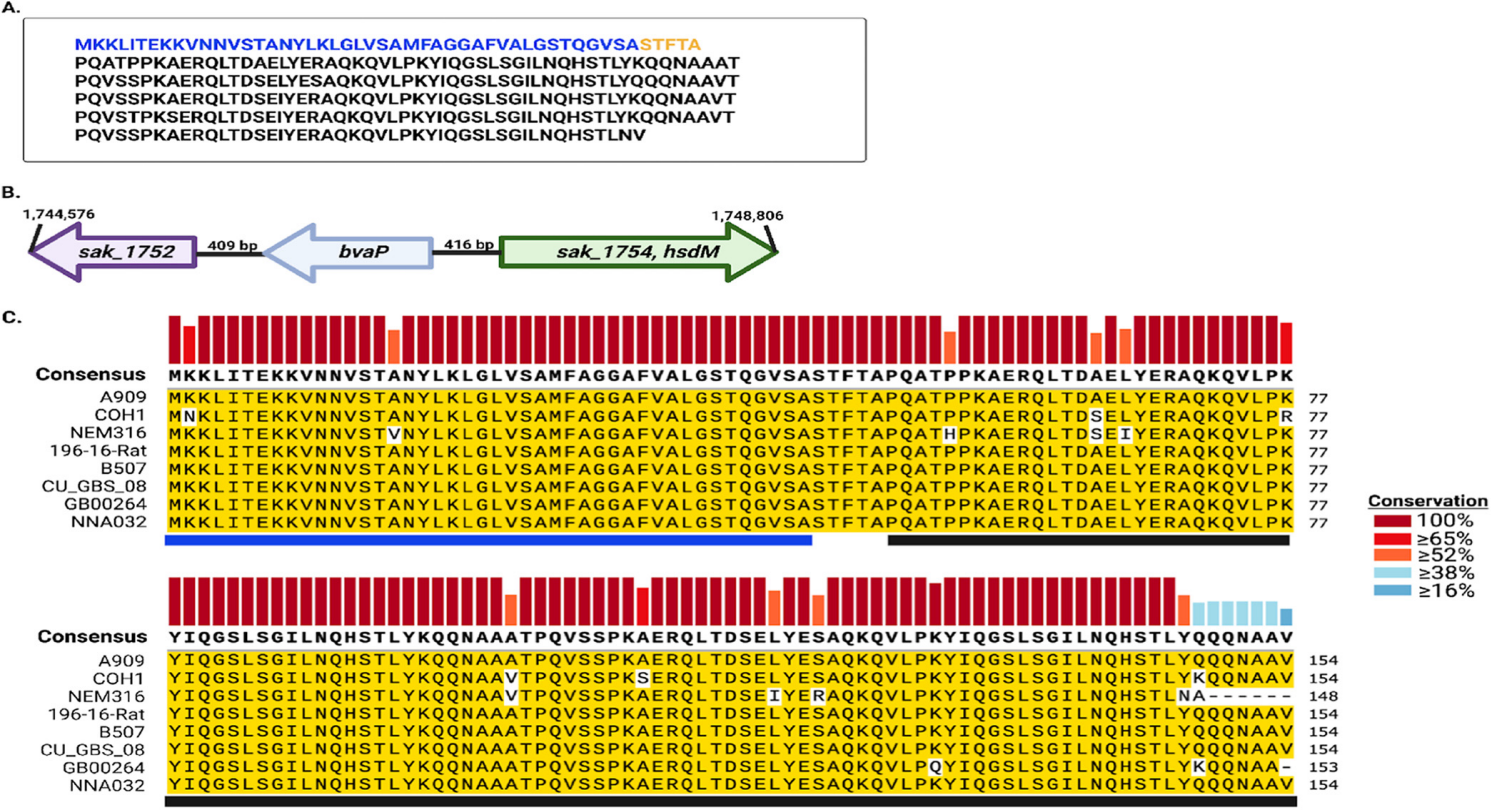
BvaP is highly conserved in GBS. (A) Protein sequence of BvaP in A909, with the putative signal sequence in blue and five repeats in black. (B) Diagrammatic representation of the location of *sak_1753*, now *bvaP*, in GBS, including the genome position for strain A909. (C) The protein sequences of BvaP homologs in 8 GBS strains were aligned using Clustal W software in SnapGene. Yellow highlighting represents conserved amino acids relative to the reference sequence from strain A909. Vertical bars on top represent residue conservation among strains. Horizontal bars below represent the putative signal sequence (blue) and the first two repeated domains (black).

BvaP (Sak_1753) from GBS strain A909 is comprised of 307 amino acids and contains almost five complete repeats of 53 amino acids. Figure 1A depicts the amino acid makeup of BvaP from A909, with the proposed signal sequence shown in blue, followed by the 5 repeated domains. *bvaP* is located more than 400 bp from the nearest gene on either side and is not predicted to be cotranscribed with neighboring genes (Fig. 1B). The putative signal sequence and the first repeated domain of BvaP were used as the query sequences in an NCBI database BLAST analysis to find all possible homologs. BvaP was found in all sequenced GBS strains isolated from several host species. The number of repeats differs between strains but generally ranges from 2 to 6. The sequence of the protein is very highly conserved within GBS strains (eight strains are shown in Fig. 1C), but homologs are also found with less conservation in a few closely related species, including Streptococcus equi.

### Deletion of *bvaP* alters GBS surface-associated phenotypes.

**FIG 2:**
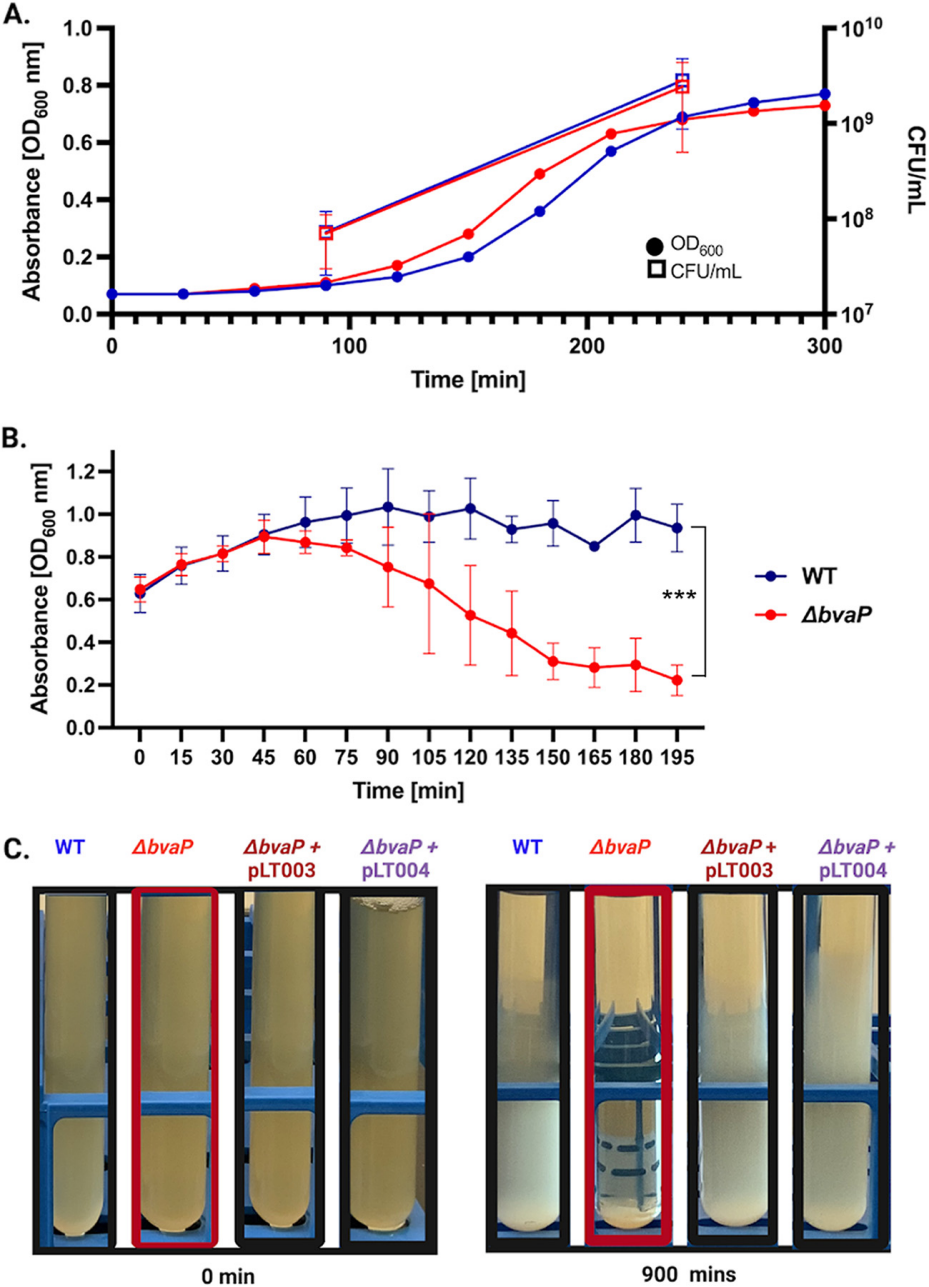
Hyperaggregation is observed in the Δ*bvaP* mutant despite normal growth kinetics and viability. (A) Growth curves of the WT (blue) and Δ*bvaP* (red) strains in THY medium, including the OD_600_ and CFU per milliliter at two points. (B) Sedimentation curves of WT and Δ*bvaP* cells or cells containing the complementation plasmids including the constitutive (pLT003) or native (pLT004) promoters in front of *bvaP*, left undisturbed for 3 h. (C) Macroscopic images of the WT, Δ*bvaP*, and complemented strains without shaking at 0 and 900 min. Two-way ANOVA was used for sedimentation rates to analyze statistical significance (***, *P* < 0.001).

The creation of a *bvaP* deletion strain was achieved by direct allelic exchange in GBS A909. Constitutive expression and complementation strains were also created using plasmids with a P*_recA_* or P*_bvaP_* promoter, respectively, in front of *bvaP*. All strains were confirmed by nucleotide sequence analysis, and quantitative reverse transcriptase real-time PCR (qRT-PCR) showed that the constitutive strain expressed *bvaP* at levels approximately 4-fold higher than those of the wild type (WT) under the same growth conditions, whereas no expression was seen in the mutant (see Fig. S1 in the supplemental material). Growth curves showed no differences in overall growth rates or viability between the mutant and WT strains in Todd-Hewitt broth supplemented with yeast extract (THY medium) (Fig. 2A); however, a difference in cell clumping was observed in samples incubated without shaking at room temperature (Fig. 2B and C). Analysis of the sedimentation rate showed significant settling during growth, with a decrease in culture turbidity over time for the mutant, while the turbidity of the WT remained relatively constant for long periods (Fig. 2B). Complementation of the *bvaP* deletion strain with plasmids either constitutively expressing *bvaP* (pLT003) or expressing *bvaP* from its native promoter (pLT004) appeared as the WT (Fig. 2C). Cell surface hydrophobicity changes have previously been linked to changes in bacterial sedimentation rates (12–14), but we observed no significant difference in surface hydrophobicity between the WT and mutant strains (Fig. S2). There were also no differences observed in hemolysis or capsule in the *bvaP* mutant and complementation strains (Fig. S2).

10.1128/msphere.00421-22.2 FIG S1 *bvaP* expression in WT and mutant cells. Reverse transcriptase real-time PCR amplification of *bvaP* was performed on cells grown to an OD_600_ of 0.4. The data shown are the averages from 4 biological replicates, each containing three technical replicates. Data are relative to the WT using *gyrA* as a control gene. **, *P < *0.01; ****, *P < *0.0001 (by ordinary one-way ANOVA). Download FIG S1, TIF file, 0.1 MB. Copyright © 2022 Thomas and Cook. 2022 Thomas and Cook. https://creativecommons.org/licenses/by/4.0/ This content is distributed under the terms of the Creative Commons Attribution 4.0 International license.

10.1128/msphere.00421-22.3 FIG S2 Loss of BvaP does not affect bacterial surface hydrophobicity, capsule production, or hemolytic activity. (A) Bacterial adhesion of the WT and Δ*bvaP* strains to hydrocarbon (*o*-xylene). An unpaired *t* test shows no significant difference between the WT and Δ*bvaP* strains. (B) Anthony direct-dry capsule staining of WT and Δ*bvaP* cells viewed at a ×1,000 magnification on an Olympus U-LHLEDC. The capsule is seen as a clear refractile area surrounding the streptococcal chains. Bar = 10 μm. (C) Hemolytic activities of WT A909, the Δ*bvaP* mutant, and the complementation construct Δ*bvaP*/pLT004 streaked onto blood agar plates. Hemolysis is indicated by a clear zone surrounding the colonies. Download FIG S2, TIF file, 0.3 MB. Copyright © 2022 Thomas and Cook. 2022 Thomas and Cook. https://creativecommons.org/licenses/by/4.0/ This content is distributed under the terms of the Creative Commons Attribution 4.0 International license.

**FIG 3:**
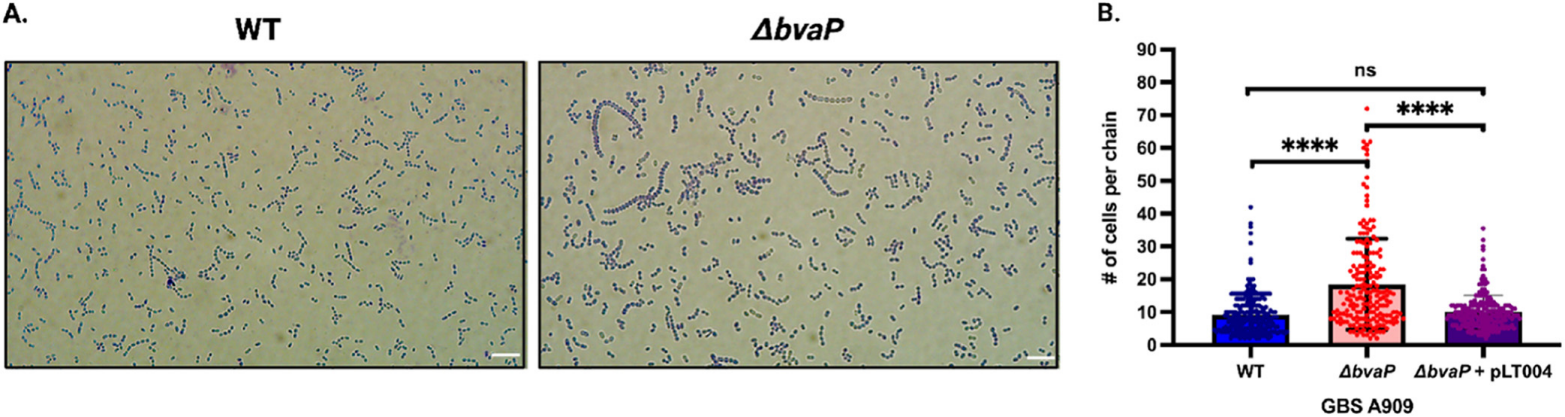
Deletion of *bvaP* increases the average chain length of GBS A909 bacterial cells. (A) Representative images of crystal violet-stained planktonic cultures grown overnight (bars = 10 μm). (B) Average number of cells per chain observed in at least 200 bacterial chains of the WT (blue), Δ*bvaP* (red), and Δ*bvaP*/pLT004 (purple) strains. Data were statistically analyzed using one-way ANOVA (****, *P* > 0.0001; ns, not significant).

To explore whether the differences in sedimentation rates were linked to cell chaining or clumping, bacteria were grown overnight, stained with crystal violet, and imaged. A909 Δ*bvaP* formed chains of a wider range of sizes, including chains much longer than those of the WT (Fig. 3A). WT, Δ*bvaP*, and native complementation (pLT004) strains were grown overnight, and ~200 chains were counted to quantify the number of individual cells in each GBS chain. On average, the Δ*bvaP* mutant had significantly long chains, with an average of 20 (±13.9) cells per chain (median value = 14.5 cells per chain), versus the WT, which averaged 9 (±6.5) cells per chain (median value = 8.0 cells per chain), and the complemented strain, which averaged 10 (±5.1) cells per chain (median value = 9.0 cells per chain) (Fig. 3B). Complementarily, analysis of log-phase cells showed the same chain length differences between the strains during log phase (data not shown).

**FIG 4:**
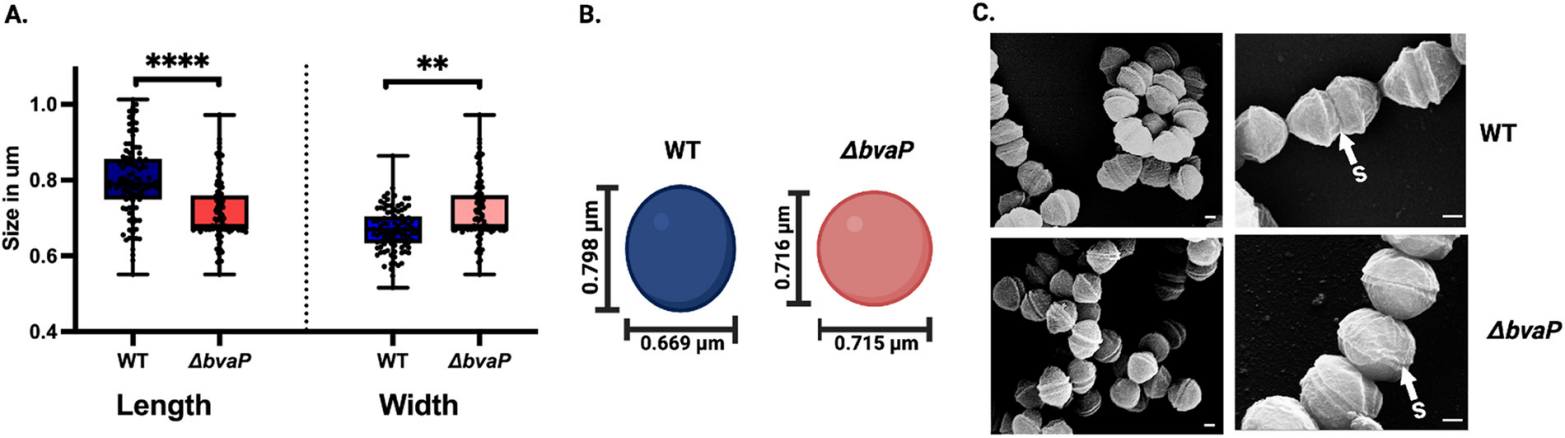
Deletion of *bvaP* causes modifications of GBS cell shape and morphology. (A) Size of GBS cells grown on a glass surface (*n* = 200). (B) Diagrammatic illustration of the WT A909 cell diameter compared to that of the Δ*bvaP* strain. (C) Representative scanning electron micrographs of WT and mutant GBS cells coated with palladium and titanium for surface morphology imaging. S represents the division septum. Bars = 0.2 μm. *P* values were determined by a Mann-Whitney U test (**, *P < *0.01; ****, *P < *0.0001).

Independent of the chain length, differences in cell size and morphology between the two strains were observed microscopically. To evaluate the morphological differences between the WT and mutant strains, scanning electron micrographs were obtained to visualize the details of the bacterial surface morphology and to measure the cell length and diameter. Interestingly, the length and width of the mutant cells were observed to be significantly different from those of the WT cells. WT cells appeared more ovoid, with an average length of 0.80 μm (±0.10 μm) and a width of 0.70 μm (±0.06 μm), while the mutant cells were more spherical, and they were shorter, at 0.72 μm (±0.10 μm), and slightly wider, at 0.72 μm (±0.08 μm) (Fig. 4A and B). Scanning electron microscopy (SEM) also exposed variations in the division septa. In the absence of BvaP, the septum is observably narrower than the WT in some cells (Fig. 4C).

### BvaP is found in both cell surface-associated and secreted fractions.

**FIG 5:**
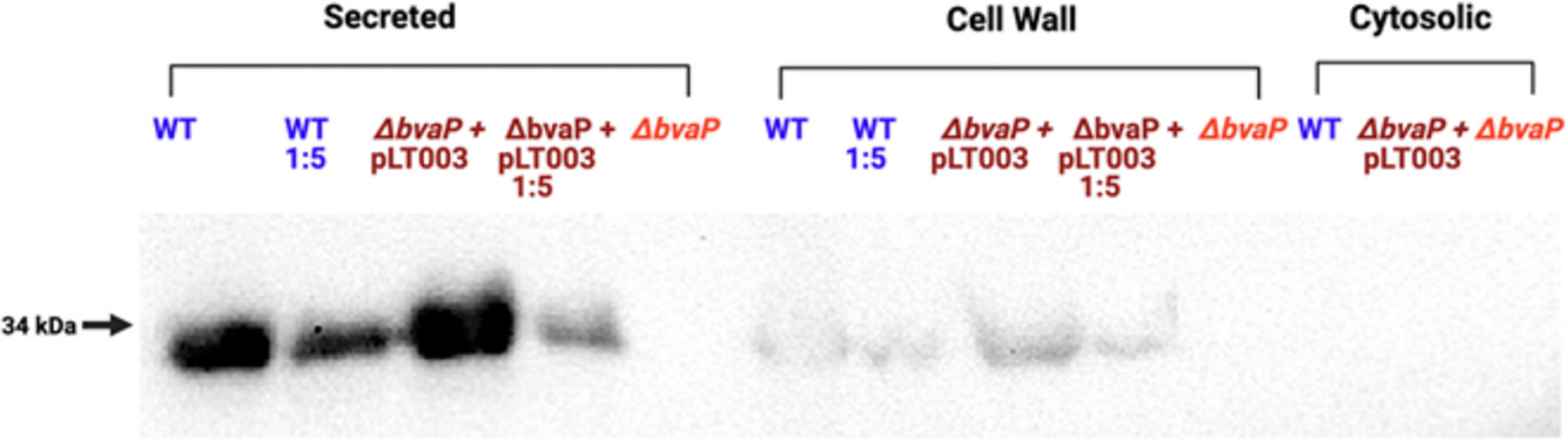
BvaP is localized at the cell surface as well as secreted. Western blot analysis was performed on whole-cell lysates from the WT, Δ*bvaP*, and Δ*bvaP*/pLT004 strains fractionated by ultracentrifugation to identify the subcellular localization of BvaP. Anti-BvaP antibody (Sino Biological) was used for detection.

Polyclonal anti-BvaP antibody against two peptides from BvaP was generated by Sino Biologicals. A cellular differential fractionation protocol was used to separate the cytosolic, membrane, and secreted fractions. Under laboratory growth conditions, BvaP is both found in the culture supernatant and localized in the cell wall fraction of the cell (Fig. 5). The amount of BvaP in the supernatant versus the amount localized in the membrane indicates that there may be multiple roles for this protein that involve being both membrane bound and secreted. The localization of at least a portion of the produced BvaP protein to the membrane of the cell was further confirmed by immunofluorescence microscopy (Fig. S3).

10.1128/msphere.00421-22.4 FIG S3 BvaP is membrane localized. Immunofluorescence microscopy of GBS cells induced with mouse vagina lavage fluid shows BvaP localized on the membrane of the cell. Wheat germ agglutinin was used as the control stain, and fluorescein isothiocyanate (FITC)-conjugated secondary antibody was used to stain BvaP-HA (hemagglutinin). Bar = 10 μm. Download FIG S3, TIF file, 0.2 MB. Copyright © 2022 Thomas and Cook. 2022 Thomas and Cook. https://creativecommons.org/licenses/by/4.0/ This content is distributed under the terms of the Creative Commons Attribution 4.0 International license.

### BvaP aids in GBS adherence to ECM components and human vaginal epithelial cells (VK2).

**FIG 6:**
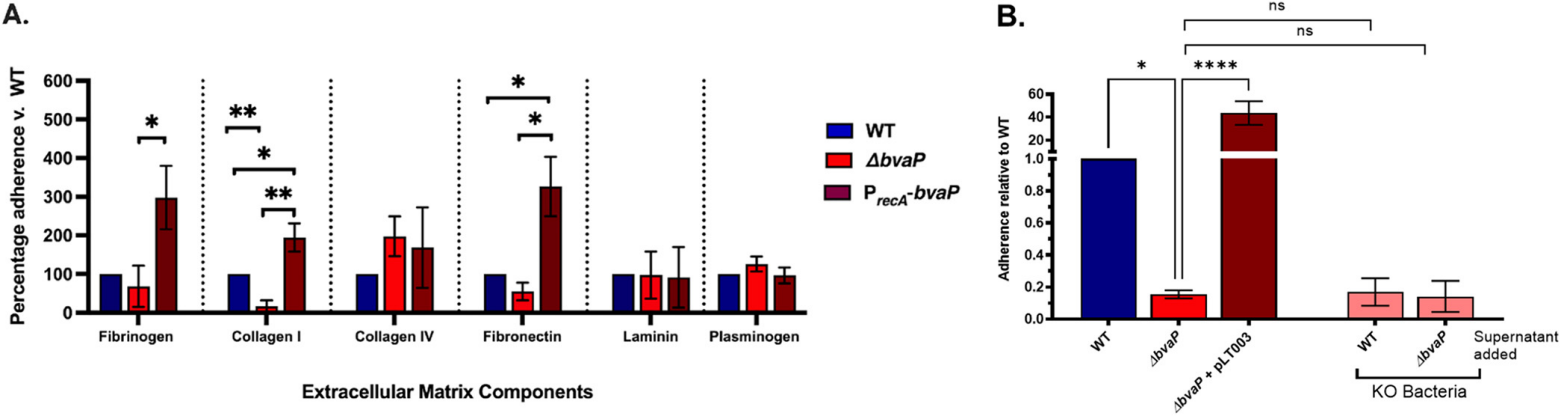
BvaP is necessary for proficient binding to some ECM components and human vaginal epithelial cells *in vitro*. (A) Adherence assays using the extracellular matrix components human fibrinogen, collagens I and IV, fibronectin, and laminin using the WT, the Δ*bvaP* strain, and the overexpression construct Δ*bvaP*/pLT003. (B) Adherence assays using human vaginal epithelial cells (VK2) at an MOI of ~10 show decreased binding of the *bvaP* mutant strain and increased binding of the strain overexpressing *bvaP*. Pink bars, Δ*bvaP* cells incubated with the WT or Δ*bvaP* supernatant prior to adherence. The addition of the WT supernatant did not result in increased binding of the Δ*bvaP* strain to VK2 cells. Assays were completed in at least technical triplicate for each data point. Averages from ≥3 biological replicates ± standard errors of the means (SEM) are shown. Statistical significance was analyzed by one-way ANOVA with a Kruskal-Wallis *post hoc* test (*, *P < *0.05; **, *P* < 0.01; ****, *P* < 0.0001; ns, not significant). KO, knockout.

GBS adherence to host cells is an important preliminary step for successful mucosal colonization. To ascertain the role of BvaP in the colonization of the host mucosa, we examined the adhesion of the WT and mutant strains to host extracellular matrix (ECM) components and a human vaginal epithelial cell line, VK2. To determine whether BvaP interacts with human ECM components, we examined the binding of our mutant GBS strains to fibrinogen, fibronectin, laminin, plasminogen, and collagens I and IV. The deletion or overexpression of *bvaP* does not affect the binding of A909 to collagen IV, plasminogen, and laminin. However, the constitutive expression of *bvaP* using the P*_recA_*-*bvaP* (pLT003) plasmid resulted in a significant increase in adherence to human collagen I, while the deletion of *bvaP* significantly reduced adherence (Fig. 6A; Fig. S4A). The constitutive expression of *bvaP* also resulted in significantly increased binding of bacteria to fibronectin and fibrinogen. Published RNA-seq data showed that the expression level of *bvaP* is low in liquid culture (10), so a comparison of the knockout and constitutive expression strains is likely more physiologically relevant to *in vivo* conditions in laboratory medium. The heterologous expression of BvaP in the related organism Streptococcus pyogenes (group A streptococcus [GAS]), which does not contain a BvaP homolog, slightly but significantly increased binding to collagen I compared to WT NZ131 (Fig. S5).

10.1128/msphere.00421-22.5 FIG S4 BvaP is needed for efficient binding *in vitro* to cultured cells and some ECM components. Shown is the absolute adherence of GBS to the extracellular matrix components human fibrinogen, collagens I and IV, fibronectin, and laminin (A) or vaginal epithelial cells (B). Adherence assays with human vaginal epithelial cells (VK2) and bacteria at an MOI of 10 show decreased binding of the *bvaP* mutant strain and increased binding of the strain constitutively expressing *bvaP*. The assay was completed in at least technical triplicate for each data point. Averages from ≥3 biological replicates ± SEM are shown. Statistical significance was analyzed by one-way ANOVA (*, *P* < 0.05; **, *P < *0.01). Download FIG S4, TIF file, 0.8 MB. Copyright © 2022 Thomas and Cook. 2022 Thomas and Cook. https://creativecommons.org/licenses/by/4.0/ This content is distributed under the terms of the Creative Commons Attribution 4.0 International license.

10.1128/msphere.00421-22.6 FIG S5 Heterologous expression of BvaP in S. pyogenes increases binding to collagen I. Shown are data from an adherence assay with collagen I using WT S. pyogenes NZ131 and NZ131 with a constitutive expression plasmid (NZ131/pLT003). The addition of BvaP to S. pyogenes increased the ability of the bacteria to bind to collagen I. The assay was completed in at least technical triplicate for each data point. Averages from ≥3 biological replicates ± SEM are shown. Statistical significance was analyzed by one-way ANOVA (*, *P < *0.05). Download FIG S5, TIF file, 0.1 MB. Copyright © 2022 Thomas and Cook. 2022 Thomas and Cook. https://creativecommons.org/licenses/by/4.0/ This content is distributed under the terms of the Creative Commons Attribution 4.0 International license.

For cell culture adherence assays, VK2 cells were grown in 24-well plates to ~70 to 80% confluence. Bacterial cells were grown to log phase (OD_600_ [optical density at 600 nm] = 0.4 to 0.5), washed with phosphate-buffered saline (PBS), and resuspended in antibiotic-free keratinocyte serum-free growth medium (KSFM) at a multiplicity of infection (MOI) of ~10. Following a 30-min incubation with bacteria, VK2 cells were washed to remove nonadherent bacteria, and the adherent cells were quantified. The constitutive expression of *bvaP* resulted in a significant increase in GBS adherence to VK2 cells, while the deletion of *bvaP* resulted in significantly decreased binding compared to the WT (Fig. 6B; Fig. S4B).

Because we observed that BvaP was present in both the membrane and the supernatant (Fig. 5), we tested whether BvaP secreted by WT cells would rescue the VK2 attachment phenotype that we observed for the Δ*bvaP* strain. Actively growing WT and Δ*bvaP* cultures were spun down, and the filtered supernatant was added back to Δ*bvaP* cultures prior to measuring adherence to VK2 cells. The addition of the WT supernatant containing secreted BvaP (Fig. 5) or the supernatant from a Δ*bvaP* culture did not return binding to WT levels (Fig. 6B), indicating that it is likely that membrane-bound BvaP is mediating binding to cultured epithelial cells.

### *In vitro* biofilm formation by GBS is inhibited in the absence of *bvaP*.

**FIG 7:**
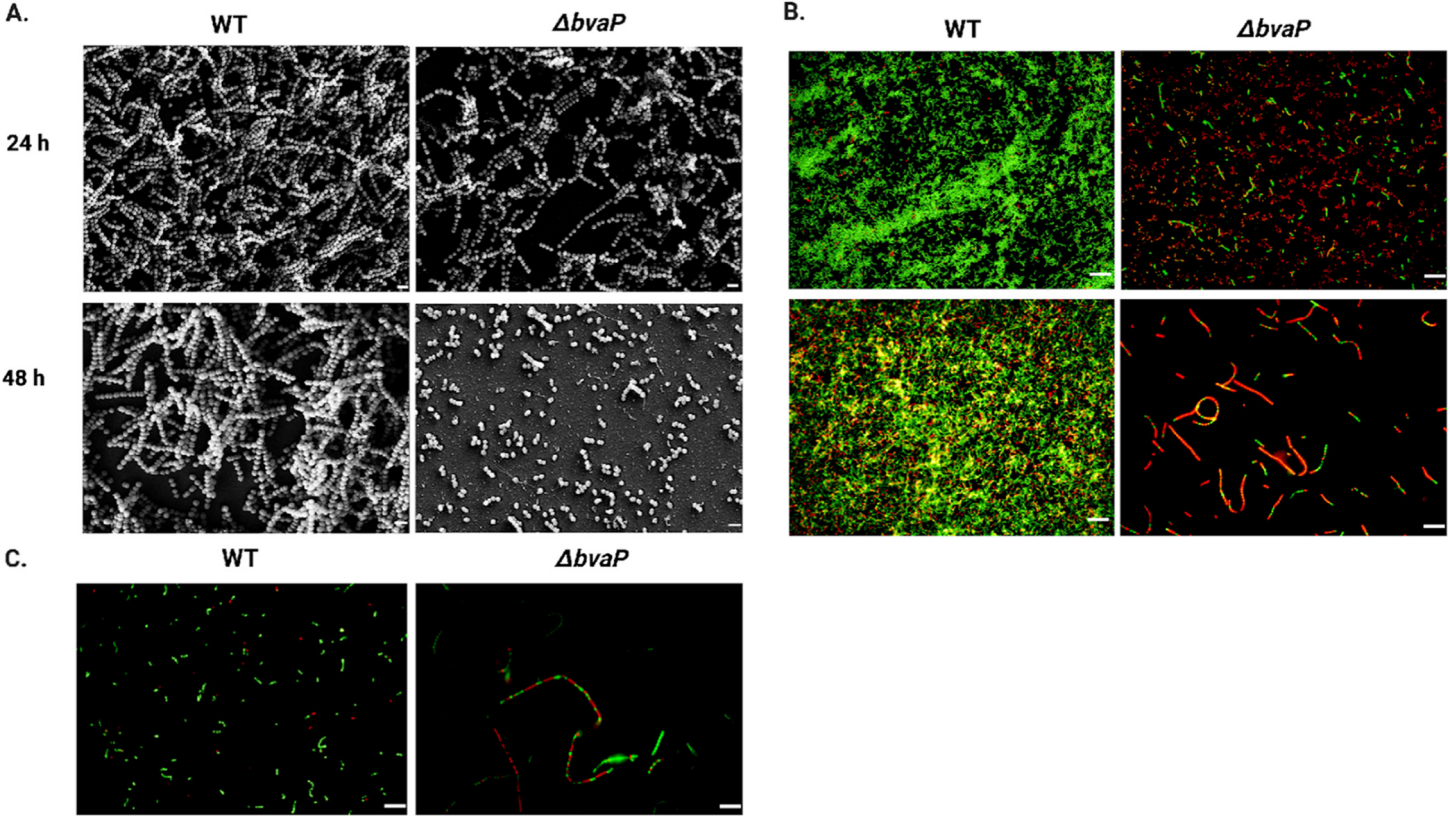
BvaP is required for efficient biofilm formation on glass surfaces. (A) Scanning electron micrographs of GBS WT and Δ*bvaP* strains. Bars = 2 μm. (B and C) BacLight live/dead staining (green, SYTO 9; red, propidium iodide) of GBS biofilms at 24 and 48 h postinoculation (B) and log-phase planktonic cells (C). Bars = 10 μm.

The formation of bacterial biofilms is often proposed to be important for the colonization of the host mucosa. To examine the impact of BvaP on *in vitro* GBS biofilm formation, 24- and 48-h biofilms were grown on glass coverslips in 6-well plates. Biofilms were examined using both SEM and live/dead staining in conjunction with fluorescence microscopy to visualize the biofilm structure. The Δ*bvaP* mutant was unable to form the structurally intricate biofilms seen in the WT samples (Fig. 7A), and the biofilm that was formed consisted of many areas without biofilm coverage and places where cells stained red with a live/dead stain, indicating increased membrane permeability (Fig. 7B). Similar staining was seen in planktonic cells (Fig. 7C), indicating some issues with membrane permeability in the Δ*bvaP* mutant. To check cell viability during biofilm growth, a solution containing 0.05% trypsin–EDTA and 0.25% Triton X-100 was used to detach biofilms from the glass, and bacteria were quantified by dilutions and CFU counts as well as crystal violet staining of the biofilm biomass. Mutant biofilm CFU counts and biomass measurements were lower than those of the WT at 24 h but not significantly, whereas there was a statistically significant difference at 48 h (Fig. S5).

### BvaP is required for efficient *in vivo* murine vaginal colonization.

**FIG 8:**
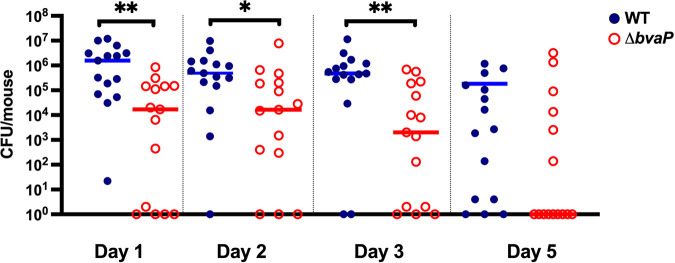
BvaP is required for *in vivo* murine vaginal colonization. Estradiol-synchronized CD-1 mice were colonized with 10^7^ CFU/mouse. The bars are set at the median values. Data are compiled from two experiments testing both strains on the same day using 10 or 5 mice per group. Data were analyzed using a Mann-Whitney test (*, *P < *0.05; **, *P < *0.01) (*n* = 15).

To evaluate the role of BvaP in GBS colonization of the vaginal tract, a murine vaginal colonization model was employed. Mice were injected intraperitoneally with β-estradiol to synchronize their estrus cycles on day −1. On day 0, mice were inoculated intravaginally with ~10^7^ CFU of bacteria. On subsequent days, vaginal washing was done, followed by plating onto GBS selective agar to quantify bacterial numbers. Compared to the WT, the Δ*bvaP* strain showed significantly less GBS colonization on days 1 to 3 and a trend toward decreased colonization on day 5, indicating an important role for this protein in vaginal adherence, especially during the first 3 days of the mucosal colonization process (Fig. 8), matching published RNA-seq expression data (10, 11).

## DISCUSSION

Previously reported transcriptomic studies identified an uncharacterized gene, *sak_1753*, as the most highly upregulated gene in GBS strain A909 when comparing growth on day 2 of vaginal colonization to growth in laboratory culture (10) and the growth of GBS strain CJB111 at day 3 of vaginal colonization (11). Here, we have begun to characterize Sak_1753, now identified as group B streptococcus vaginal adherence protein (BvaP). While BvaP is found and highly conserved in all sequenced strains of GBS (Fig. 1), few homologs are present, even in closely related species such as S. pyogenes. No known protein family or domain homologs were identified using Pfam and Phyre protein prediction software. Here, we present the first information on the effects of BvaP on GBS surface morphology and phenotypes associated with vaginal colonization.

Initial observations indicate that the deletion of *bvaP* from the A909 genome increases cell aggregation in laboratory medium (Fig. 2) and changes the overall cell shape, resulting in bacteria that are significantly shorter and wider than WT bacteria (Fig. 4). Additionally, the chains formed by Δ*bvaP* mutants are significantly longer than those formed by the WT (Fig. 3), potentially denoting incomplete cell division. Bacterial cell shape and chain length changes in particular mutants or under particular growth conditions have been reported for many chain-forming bacteria. For streptococci, the regulation of chain length has been reported to be dependent on cell wall-associated autolytic activity and the presence of autolysins (15–17) as well as environmental factors such as pH (18), growth medium (19), and salt concentrations (15), among others. There have been studies linking chain length in various streptococci with pathogenesis, showing that long-chain cells may promote adherence and colonization due to an increased surface area, which allows multivalent adhesive interactions (19), while short chains are associated with more invasive diseases such as meningitis (20). Dalia and Weiser described that an increase in the S. pneumoniae chain length in a mutant strain correlated with an increase in cell death through C3-mediated neutrophil opsonophagocytosis (20). Alternatively, other studies show that longer-chain phenotypes in mutants are associated with decreased biofilm formation and adherence and colonization abilities, like what is seen in our studies with BvaP (21, 22).

The reduced ability to form biofilms is also often linked to phenotypes such as increased aggregation and chain length. A mutant in biofilm regulatory protein A (BrpA) from GBS caused both a chain length increase and a biofilm defect (23). Similarly, the deletion of *bvaP* was correlated with a decrease in biofilm formation and an increase in the chain length increase in our study (Fig. 7). Biofilm formation by the Δ*bvaP* strain at 24 h was somewhat reduced compared to that of the WT (Fig. 7; see also Fig. S6 in the supplemental material), and this difference was enhanced significantly by 48 h, where a noticeable biofilm defect was seen in the Δ*bvaP* strain microscopically (Fig. 7) and by CFU counts and crystal violet staining (Fig. S6) compared to the WT.

10.1128/msphere.00421-22.7 FIG S6 Biofilms formed by *bvaP* mutants on glass are less viable and less dense after 48 h than WT biofilms. (A) Twenty-four-hour and 48-h biofilms grown in 6-well plates on coverslips at 37°C in 5% CO_2_ were detached with trypsin-EDTA and Triton X-100 and dilution plated onto THY medium with Spec to quantify viable CFU per milliliter. (B) Crystal violet staining of biofilms at 24 h postinoculation. *, *P < *0.05; **, *P < *0.01; ****, *P* < 0.0001 (by one-way ANOVA) (*n* = 3). Download FIG S6, TIF file, 0.2 MB. Copyright © 2022 Thomas and Cook. 2022 Thomas and Cook. https://creativecommons.org/licenses/by/4.0/ This content is distributed under the terms of the Creative Commons Attribution 4.0 International license.

Bacterial cell surface hydrophobicity is an important element for bacterial aggregation and surface attachment. Alterations in surface hydrophobicity have been associated with changes in biofilm formation (24), cell surface properties such as cell shape (25), and sedimentation (26). We found no change in cell surface hydrophobicity in the Δ*bvaP* mutant, suggesting that the overall cell surface chemistry remains unchanged in the absence of BvaP (Fig. S2A). Capsular alterations may be associated with aggregative phenotypes, displaying changes in chain length and adherence (27), but we did not observe differences in capsule between our WT and mutant cells (Fig. S2B). In addition, no change was seen in hemolysis between these strains (Fig. S2C).

Binding to and colonization of the host mucosa often involve interactions between bacteria and host extracellular matrix (ECM) components. Proteins expressed by streptococci can mediate interactions with host ECM components to contribute to their adherence, colonization, invasion, and evasion of host defenses (28). In the vaginal tract, collagens, especially collagens I and III; elastin; and glycosaminoglycans are predominant ECM components (29). Proteomic analyses of cervicovaginal fluids showed the presence of fibrinogen precursors, indicating that fibrinogen binding could be important for vaginal adherence by GBS (30, 31). Proteins involved in binding to specific ECM components in GBS have been identified. Examples of previously described adherence interactions include PilA binding to collagen (32); FbsA, FbsB, and serine-rich repeat (Srr) proteins mediating GBS-fibrinogen binding (7, 33, 34); and PbsP binding to plasminogen and vitronectin (35, 36). Some GBS adhesins have been shown to have dual functions in addition to binding. For example, Lmb binds laminin (8) and also aids in zinc acquisition (37), and C5a peptidase mediates interactions with fibronectin and has specific protease functions (38, 39).

Previous studies have described conflicting results as to whether GBS binds collagen I to mediate host attachment. Dramsi et al. argued that GBS strain A909 does not bind to collagen I but instead binds to fibrinogen to mediate attachment to the host (40), yet a conflicting report (32) identified significant binding to collagen I. Experiments in the former study were conducted using rat tail collagen I, compared to the latter study, which used human collagen, which may explain the differences in the findings. In this study, we found that GBS A909 binds to human collagen I and that this interaction is, at least partially, mediated by BvaP (Fig. 6A). We observed GBS binding to both fibrinogen and fibronectin (Fig. 6A), and these interactions also appear to be affected by the presence or amount of BvaP. Furthermore, we investigated the effects of the expression of BvaP in another streptococcal species, S. pyogenes, and found a slight but significant increase in binding to collagen I when BvaP was constitutively expressed compared to the WT (Fig. S5). This suggests that BvaP potentially increases adherence when expressed heterologously in related species that do not contain BvaP homologs. In addition to specific ECM components, we also undertook adherence assays using immortalized cultured vaginal epithelial VK2 cells. Cells in which *bvaP* was deleted had a greatly decreased ability to bind VK2 cells, and this adherence defect could be complemented by the addition of *bvaP* in *trans* (Fig. 6B).

It could be postulated that cellular morphological changes alter bacterial binding to ECM components and the host. We find that the changes in ECM binding appear to be specific rather than generalized, as the deletion or constitutive expression of BvaP does not affect adherence to all components tested. Further research is under way to determine whether BvaP directly binds to ECM components and/or if alterations in cell surface properties influence this binding.

The results of our murine vaginal colonization assays agree with our *in vitro* data indicating a role for BvaP in host binding. Mice inoculated with the Δ*bvaP* strain were colonized significantly less over the 5-day period than mice inoculated with the WT. Since our data indicate that BvaP is important for binding to some ECM components, it would be interesting to examine if the ability of BvaP to adhere to the host vaginal mucosa is dependent on interactions with these ECM components specifically or is due to overall changes in cell surface properties affecting colonization more generally.

BvaP was not detected in the bacterial cytosol but was found both on the cell surface and in the extracellular space (Fig. 5). The detection of BvaP at the cell surface suggests that BvaP is potentially anchored in the cell wall or cell membrane in some way. In Gram-positive bacteria, including GBS, proteins may be covalently anchored to the cell wall through a highly conserved carboxy-terminal motif, Leu-Pro-X-Thr-Gly (LPXTG), which is cleaved and then attached to an amino group of the processed protein in the peptidoglycan via sortase (41). Less commonly, proteins are tethered to an amino-terminal lipid-modified cysteine (42, 43). We are currently exploring the mechanism by which BvaP, which lacks a canonical LPXTG motif, is tethered to the surface or released extracellularly, either actively or passively. Western blotting (Fig. 5) and immunofluorescence microscopy (Fig. S3) indicate that at least a portion of the BvaP produced is found located on the cell surface, but staining does not demonstrate an obvious localization of the protein at particular sites such as the division septum, and staining was not consistent throughout the population. Further analysis of protein localization *in vivo* will be needed to determine whether BvaP is expressed equally on all cells and whether it is primarily membrane localized, secreted, or both, *in vivo* in the vaginal tract.

It is possible that BvaP is actively secreted from the cells or cleaved from a membrane-bound form to release the protein into the extracellular space. It is currently unclear whether the membrane-bound protein serves a different function for the cell than the secreted protein. Adhesins are generally assumed to be surface associated, so the observation that a large portion of BvaP is secreted (Fig. 5) is a surprising result. Several hypotheses exist, which we are currently exploring. Secreted BvaP could directly affect interactions between GBS cells affecting the ability of the cells to clump or form biofilms, thus impacting the ability to bind to ECM components and mammalian cells. Additionally, or alternatively, the secreted protein could have a different function in the cell that is unrelated to the adherence phenotypes that we observe.

Seeing an adherence phenotype for a secreted protein is not unprecedented. A family of secreted proteins in Staphylococcus aureus called secretable expanded-repertoire adhesive molecules (SERAMs) are released from the cell but can still mediate adherence to host cells and ECM components (44, 45). One more well-studied SERAM, Eap, has tandem-repeat domains, mediates bacterial clumping, and interacts with ECM components such as collagen I, similarly to BvaP (46, 47). Also similarly to BvaP, a portion of Eap is observed to be associated with the membrane, while a large portion is also observed in the extracellular space. At least a subset of Eap that is released rebinds to the S. aureus surface (48), which is presumed to affect the adherence of the bacteria to the host. Secreted Eap has additional roles in staphylococcal infection via immune modulation (47). It is possible that BvaP acts in a similar way or that secreted BvaP has additional roles in GBS colonization or infection other than adherence. Further studies will be needed to determine the potentially varied roles of surface-associated and secreted BvaP.

Collectively, our data provide an important initial characterization of a previously hypothetical protein, Sak_1753, now BvaP, conserved throughout GBS strains. BvaP is integral to maintaining the cell morphology, chain length, and *in vitro* biofilm formation ability of GBS and is required for efficient adherence to vaginal epithelial cells, both in *in vitro* adherence assays and in *in vivo* murine vaginal colonization experiments. It is possible that these adherence properties are mediated by ECM components such as collagen I, fibrinogen, and fibronectin, although future studies are needed to determine whether these are direct interactions between membrane-bound BvaP and ECM components and the role of these interactions *in vivo*.

More research will be needed to determine whether the colonization defect observed in the Δ*bvaP* mutant is related specifically to chain length, cell surface property alterations, host immune interactions, specific adhesion binding, or some combination of these. As BvaP is very highly expressed in the vaginal tract (10, 11) and conserved among all sequenced GBS strains, it has the potential to serve as a GBS protein-based vaccine candidate. Further experiments to determine whether secreted BvaP, surface-associated BvaP, or both are important for binding to the host and whether BvaP elicits a strong immune response *in vivo* are under way. Additional studies on the repeated domains of BvaP that vary between strains will also uncover more details about the mechanism behind BvaP’s control of colonization phenotypes.

## MATERIALS AND METHODS

### Bacterial strains, media, plasmids, primers, and growth conditions.

All strains and plasmids are shown in Table S1 in the supplemental material, and the primers used in this study are outlined in Table S2. Supplemental Materials and Methods are found in Text S1. All GBS strains used in this study were derived from the clinical isolate A909 (GenBank accession number NC_007432). GBS strains were routinely grown in Todd-Hewitt medium (BD Bacto) supplemented with 0.2% (wt/vol) yeast extract (Fisher Scientific) (THY medium) statically at 37°C. Plating was done on THY agar or CHROMagar StrepB agar (DRG). GBS was grown at 37°C unless it was transformed with a temperature-sensitive plasmid, in which case the growth temperature was 30°C. When necessary, the following antibiotics were included at the indicated concentrations for GBS propagation: erythromycin (Erm) at 0.5 μg mL^−1^, spectinomycin (Spec) at 100 μg mL^−1^, and chloramphenicol (Cm) at 3 μg mL^−1^. All Escherichia coli strains were cultivated in Luria-Bertani (LB) medium or on LB agar. E. coli was grown at 37°C with shaking unless it was transformed with a temperature-sensitive plasmid, in which case the growth temperature was 30°C. When necessary, the following antibiotics were included at the indicated concentrations for E. coli propagation: Erm at 300 μg mL^−1^, Spec at 100 μg mL^−1^, and Cm at 10 μg mL^−1^.

10.1128/msphere.00421-22.8 TABLE S1 Strains and plasmids. Download Table S1, DOCX file, 0.02 MB. Copyright © 2022 Thomas and Cook. 2022 Thomas and Cook. https://creativecommons.org/licenses/by/4.0/ This content is distributed under the terms of the Creative Commons Attribution 4.0 International license.

10.1128/msphere.00421-22.9 TABLE S2 Primers. Download Table S2, DOCX file, 0.01 MB. Copyright © 2022 Thomas and Cook. 2022 Thomas and Cook. https://creativecommons.org/licenses/by/4.0/ This content is distributed under the terms of the Creative Commons Attribution 4.0 International license.

### Creation of mutant and complemented strains.

The Δ*bvaP* mutagenesis cassette consists of the spectinomycin resistance (Spec^r^) gene flanked by 1,000 bp upstream and 996 bp downstream surrounding *bvaP*. The upstream and downstream fragments were PCR amplified from template A909 genomic DNA (gDNA) using primer pair LC206/LC244 for the upstream region and primer pair LC248/LC249 for the downstream region. The Spec^r^ gene was PCR amplified from pJC303 using primer pair LC246/LC247. The three fragments were cloned into the pMB*sacB* plasmid using 2× Hifi master mix (catalog number M5520AA; New England BioLabs [NEB]) according to the manufacturer’s instructions, creating the plasmid pLT001.

Electrocompetent GBS cells were prepared using the sucrose-free method as previously described (49), with the following modifications: electrocompetent GBS cells were grown with 1% glycine instead of 2.5%, and the sucrose concentration was increased to 0.75 M for counterselection. Transformants were propagated in THY medium containing Erm and Spec, and sucrose sensitivity was confirmed by plating serial dilutions onto THY agar with 0.75 M sucrose grown at 30°C. Single-crossover intermediates were selected after a temperature shift followed by passaging in sucrose to select for a double crossover and plasmid excision (49). The final knockout strain, A909 Δ*bvaP*::Spec^r^, was sequenced to confirm gene replacement.

For plasmid complementation under the control of the native promoter, the *bvaP* gene, including its predicted promoter region, was amplified from A909 gDNA by high-fidelity Phusion PCR using primer pair LT016/LT017. The pLZ12Spec plasmid backbone was amplified using primer pair LT011/LT012. To construct a strain constitutively expressing *bvaP*, primer pair LT005/LT009 was used to amplify the pJC303 backbone, including the P*_recA_* promoter, and primer pair LT007/LT008 was used to amplify *bvaP* from A909 gDNA. Gibson assembly for each plasmid was done using 2× Hifi master mix as described above, creating the complementation plasmid pLT004 (P*_bvaP_ bvaP*) and the constitutive plasmid pLT003 (*P_recA_ bvaP* Cm^r^). Plasmids were electroporated into electrocompetent E. coli DH5α cells. Plasmid DNA was purified using the GenElute HP plasmid miniprep kit (Krackeler) according to the manufacturer’s instructions. To switch the spectinomycin resistance gene in pLT004 with the chloramphenicol resistance gene, the *catR* gene was amplified with primer pair LT118/LT119, and the pLT004 Spec^r^ plasmid backbone was amplified with primer pair LT116/LT126. The final plasmid, pLT004 (P*_bvaP_ bvaP* Cm^r^), was assembled via Gibson assembly and propagated in E. coli as described above. Plasmids were electroporated into the A909 Δ*bvaP*::Spec^r^ strain. The final constructs were confirmed by sequencing. For experiments in group A Streptococcus (GAS), pLT003 was electroporated into electrocompetent GAS strain NZ131 and propagated under the same conditions as the ones used for GBS.

### Growth rate analysis.

Cultures grown overnight were diluted 1:20 into fresh THY medium and grown to mid-log phase (OD_600_ = 0.4 to 0.5). Cultures were diluted to an OD_600_ of 0.05 in fresh THY medium. For OD_600_ measurements, 200 μL of each culture was added to a 96-well microplate in 4 technical replicates. Replicates of THY medium only were included as a negative control. The Tecan HP Infinite 200 Pro spectrophotometer was programmed by Tecan i-control software for incubation at 37°C and measurement of the OD_600_ every 30 min with shaking for 10 s prior to measurement. Replicates were averaged and plotted as the OD_600_ versus time. For the calculation of cell viability, samples were removed at OD_600_ values of 0.2 and 0.7, and dilutions were plated onto THY agar plates to quantify CFU per milliliter.

### Cell aggregation assay.

Aggregation assays were undertaken as previously described (50). Cultures grown overnight were diluted 1:10 in 5 mL fresh THY medium and incubated at 37°C until the OD_600_ reached 0.4 to 0.6. Cells were pelleted by centrifugation and then resuspended to a final OD of 0.05 in 50 mL fresh THY medium. Cultures were grown at 37°C until the OD_600_ reached 0.4 to 0.6, vortexed, and placed on a benchtop to settle at room temperature. One milliliter of the culture was removed from just below the medium meniscus every 15 min to measure the OD_600_ using a spectrophotometer.

### Biofilm formation.

Cultures grown overnight were diluted 1:20 into fresh THY medium and allowed to grow to an OD_600_ value of ~0.4. Sterile 9-mm coverslips were added to a 6-well polystyrene microplate, which was inoculated with 1 mL of culture. Plates were incubated statically at 37°C with 5% CO_2_ for 24 h to allow cells to attach to the coverslip. The medium was aspirated and replaced with fresh THY medium, and the mixture was incubated for another 24 h. At 24 or 48 h, to quantify GBS biofilm formation, wells were washed twice with 1 mL PBS to remove cells not bound to the coverslip. Following the washes, the coverslips were removed from the wells and placed into a clean 6-well plate. The coverslips were mounted onto a microscope slide with 5 μL of live/dead stain (6 μM SYTO 9 stain and 30 μM propidium iodide) from the Live/Dead BacLight bacterial viability kit (catalog number L13152; Fisher). Images were captured at a magnification of ×1,000 with an epifluorescence microscope.

### Scanning electron microscopy.

Biofilms grown as described above for 24 and 48 h were fixed by flooding the coverslips in fixation solution (2% glutaraldehyde solution, 2% formaldehyde, 150 mM sodium cacodylate buffer, 4% sucrose, 0.15% alcian blue) for 16 h at room temperature. Samples were dehydrated by incubation for 10 min each in increasing concentrations of ethanol (50, 70, 80, 95, and 100%) and chemically dried with hexamethyldisilazane (HMDS) overnight in a fume hood (51, 52). The samples were coated with a continuous, conductive, thin-film layer of a palladium-platinum alloy prior to their visualization using the Zeiss Supra 55 microscope. SEM images (magnification, ×50,000) were used to measure the length and width of 200 GBS cells using ImageJ software. Data were analyzed by an unpaired *t* test using GraphPad Prism 9 software.

### Quantification of GBS A909 chain length.

Ten microliters of a culture grown overnight was placed onto a glass slide with a coverslip and viewed at a ×1,000 magnification on an Olympus U-LHLEDC. Images of randomly selected visual fields from two separate experiments were captured using an attached Olympus DP74 camera. At least 100 chains from each set of images were manually counted, for a total of at least 200 chains counted for each strain, as previously described (53, 54). Data were analyzed by one-way analysis of variance (ANOVA) using GraphPad Prism 9 software.

### Fractionation of membrane proteins.

Fractionation and trichloroacetic acid (TCA) precipitation were done as previously described (55, 56). An equivalent of 10 mL of the culture at an OD_600_ of 0.6 was pelleted, and the supernatant was collected. The pellet was resuspended in protoplast buffer (1 M sucrose, 60 mM Tris-HCl, 20 μg/mL lysozyme, and 100 U/mL mutanolysin). Following incubation for 45 min at 37°C, samples were pelleted, and the cell wall fraction (supernatant) was moved to a clean microcentrifuge tube. TCA was added to the supernatant samples to give a 10% final solution, and the samples were then incubated on ice for 30 min. The samples were pelleted by centrifugation at maximum speed for 15 min at 4°C. The supernatant was discarded, and the pellet was washed once with 500 μL of ice-cold acetone and once with 500 μL of ice-cold TCA wash (70% ethanol, bromophenol blue). The pellets were dried using a SpeedVac centrifuge for 10 min and resuspended in 50 μL 2× Laemmli loading buffer (4% SDS, 20% glycerol, 0.004% bromophenol blue, 0.125 M Tris-Cl [pH 6.8], 10% 2-mercaptoethanol). The protoplasts (pellet) samples were resuspended in lysis buffer (0.5 M EDTA, 0.1 M NaCl [pH 7.5]) and sonicated (85% amplitude, 10 s on and 15 s off) for 3 min. Samples were centrifuged using an S120-AT2 rotor in a Thermo Scientific Sorvall mTX150 microultracentrifuge at 4°C for 1 h at 100,000 × *g*. The supernatant containing the cytosolic fraction was also TCA precipitated, and the pellet containing the membrane and insoluble fractions was resuspended in 2× Laemmli loading buffer. Fractions were subjected to SDS-PAGE and Western blot analysis as described below.

### Western blot analysis.

Proteins were run on an SDS-PAGE gel (11% resolving, 6% stacking), probed with anti-BvaP antibody (Sino Biologicals), and visualized using an ECL kit according to the manufacturer’s instructions (catalog number 80197; Thermo Scientific). The primary antibody was diluted to 0.1 mg/mL, and anti-rabbit IgG horseradish peroxidase (HRP)-linked secondary antibody was diluted 1:1,000 (catalog number 7074S; Cell Signaling Technology).

### Extracellular matrix component adherence assay.

Human collagen I (catalog number CC050; MilliporeSigma), human plasma fibrinogen (catalog number 341576; Fisher), human Glu-plasminogen (catalog number PIRP43078; Fisher Scientific), and human plasma fibronectin (catalog number FC010; MilliporeSigma) were diluted to a working concentration of 10 μg/mL in 30% ethanol and added to wells of a 24-well plate. The plates were left to dry overnight in a laminar flow hood. Precoated human laminin (catalog number ECM103; MilliporeSigma) and collagen type IV (catalog number ECM105; MilliporeSigma) strip wells were rehydrated by incubating the wells with PBS for 15 min at room temperature. Cultures of GBS grown overnight were diluted 1:20 into fresh THY medium and grown to an OD_600_ of 0.4 to 0.6. Coated wells were washed once with PBS, and 100 μL (strips) or 200 μL (24-well plate) of the bacterial culture was added to each well. Strips and plates were incubated for 1 h at 37°C with 5% CO_2_ and then gently washed three times with PBS to remove nonadherent cells. For quantification, 100 μL (strips) or 200 μL (24-well plate) 0.2% crystal violet in 10% ethanol was added, and the mixture was incubated for 5 min at room temperature. Strips and plates were then washed three times with PBS, 200 μL solubilization buffer (50:50 mixture of 0.1 M NaH_2_PO_4_ [pH 4.5] and 50% ethanol) was added, and the mixture was incubated for 5 min at room temperature. The absorbance was measured at 570 nm on a Tecan Infinite Pro spectrophotometer microplate reader. Cultures of WT NZ131 and NZ131/pLT003 grown overnight were diluted 1:20 and grown to an OD_600_ of 0.4 to 0.5 as described above for GBS. Adherence to collagen I was measured as described above.

### VK2 adherence assay.

Cellular adherence assays were done as previously described (57). VK2 vaginal epithelial cells were cultured in 24-well tissue culture plates in keratinocyte serum-free growth medium (KSFM; Gibco) supplemented with human recombinant epidermal growth factor (rEGF) and bovine pituitary extract (BPE) plus 2% penicillin-streptomycin (Gibco) at 37°C in 5% CO_2_. Cells were grown to 70 to 80% confluence (~10^5^ cells/well) with antibiotics up to 24 h before the assay, when the medium was switched to antibiotic-free KSFM with supplements. Immediately before the assay, the eukaryotic cells were washed once with PBS. Cultures of GBS grown overnight were diluted 1:20 into fresh THY medium, grown to an OD_600_ of 0.4 to 0.5 (~1 × 10^8^ CFU/mL), washed once with PBS, and resuspended in 5 mL KSFM medium. The bacteria were added to the eukaryotic cells at an MOI of ~10:1 and incubated for 30 min at 37°C. Nonadherent bacteria were removed by three washes per well with 1 mL PBS each. Cells were then removed from the wells by incubation with 500 μL of a solution containing 0.05% trypsin–EDTA and 0.25% Triton X-100 for 5 min at 37°C with 5% CO_2_, followed by vigorous pipetting. Bacterial counts in each sample were determined by dilution plating onto THY agar plates.

To determine if BvaP secreted from WT cells would rescue mutant adhesion to vaginal epithelial cells, a supernatant add-back assay was conducted. Cells were grown and prepared as described above, with one modification: the WT and Δ*bvaP* culture supernatants were collected and passed through a 0.22-μm polyethersulfone (PES) membrane filter unit. The filtered spent supernatant was then used to resuspend bacterial pellets prior to performing the adherence assay as described above. Each adherence experiment was performed in technical triplicate or quadruplicate on a single plate, and values were averaged. The results shown are the averages from at least three biological replicates. Data were analyzed by one-way ANOVA with a Kruskal-Wallis *post hoc* test using GraphPad Prism 9 software.

### Mouse model of vaginal colonization.

Female outbred CD-1 mice (Charles River) aged 6 to 8 weeks were used for all experiments. Experiments were performed as previously described (10, 58). Briefly, 1 day prior to inoculation (day −1), mice were given an intraperitoneal injection of 0.5 mg β-estradiol valerate (Alfa Aesar) suspended in 100 μL filter-sterilized sesame oil (Acros Organics MS) to synchronize estrus. On day 0, mice were vaginally inoculated with 10 μL bacteria grown to an OD_600_ of 0.4 and resuspended in PBS, at a concentration of ~1 × 10^9^ CFU/mL. On days 1, 2, 3, and 5, the vaginal lumen was washed with 50 μL sterile PBS using a pipette to gently circulate the fluid approximately 6 to 8 times. The lavage fluid was then collected and placed on ice for no more than 30 min. The vaginal lavage fluid was serially diluted in PBS and plated onto CHROMagar StrepB to obtain CFU counts. Experiments were performed in biological duplicate on groups of 5 or 10 mice at a time. Murine colonization studies were reviewed and approved by Binghamton University Laboratory Animal Resources (LAR) and by the Binghamton Institutional Animal Care and Use Committee (IACUC) under protocol numbers 803-18 and 857-21. 

10.1128/msphere.00421-22.1 TEXT S1 Supplemental methods. Download Text S1, DOCX file, 0.02 MB. Copyright © 2022 Thomas and Cook. 2022 Thomas and Cook. https://creativecommons.org/licenses/by/4.0/ This content is distributed under the terms of the Creative Commons Attribution 4.0 International license.
