# Supplementary figures and images for: A Novel Conserved Protein in Streptococcus agalactiae, BvaP, Is Important for Vaginal Colonization and Biofilm Formation
Source: mSphere. 2022 Oct 11;7(6):e00421-22. doi: 10.1128/msphere.00421-22 (PMC9769775; doi:10.1128/msphere.00421-22)

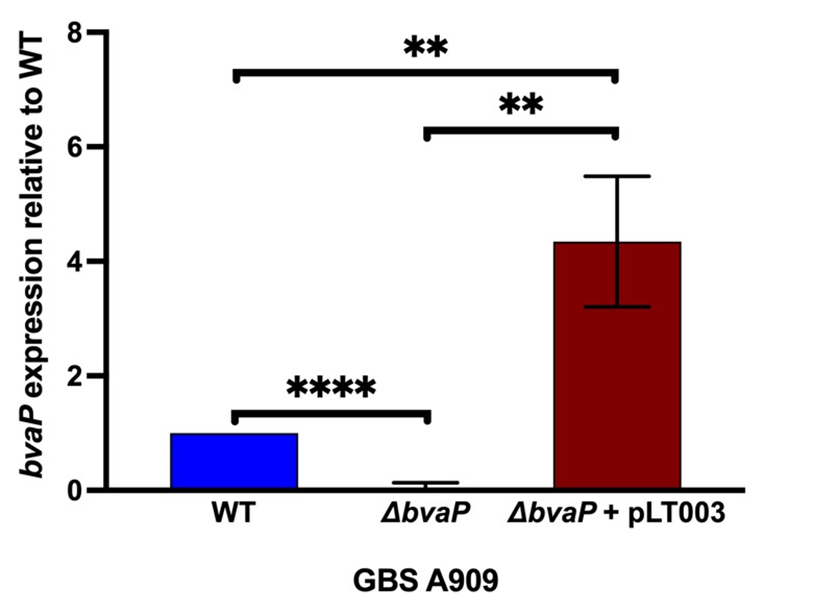

Supplement: FIG S1 [file msphere.00421-22-s0001.tif]

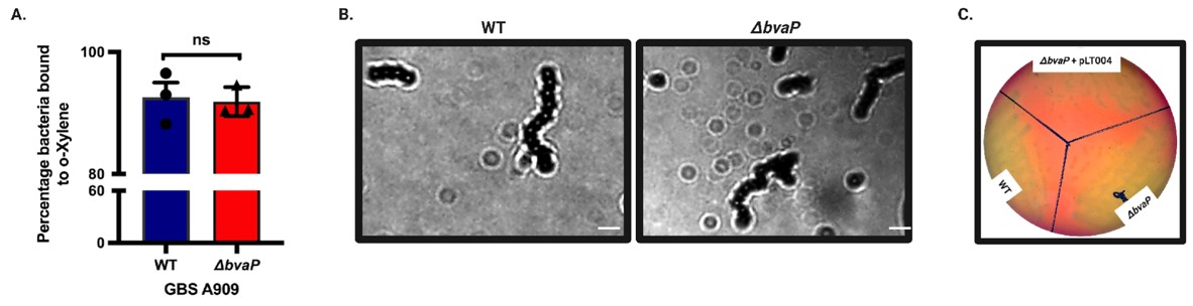

Supplement: FIG S2 [file msphere.00421-22-s0002.tif]

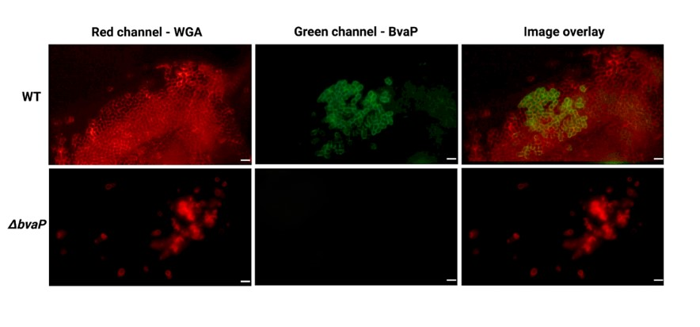

Supplement: FIG S3 [file msphere.00421-22-s0003.tif]

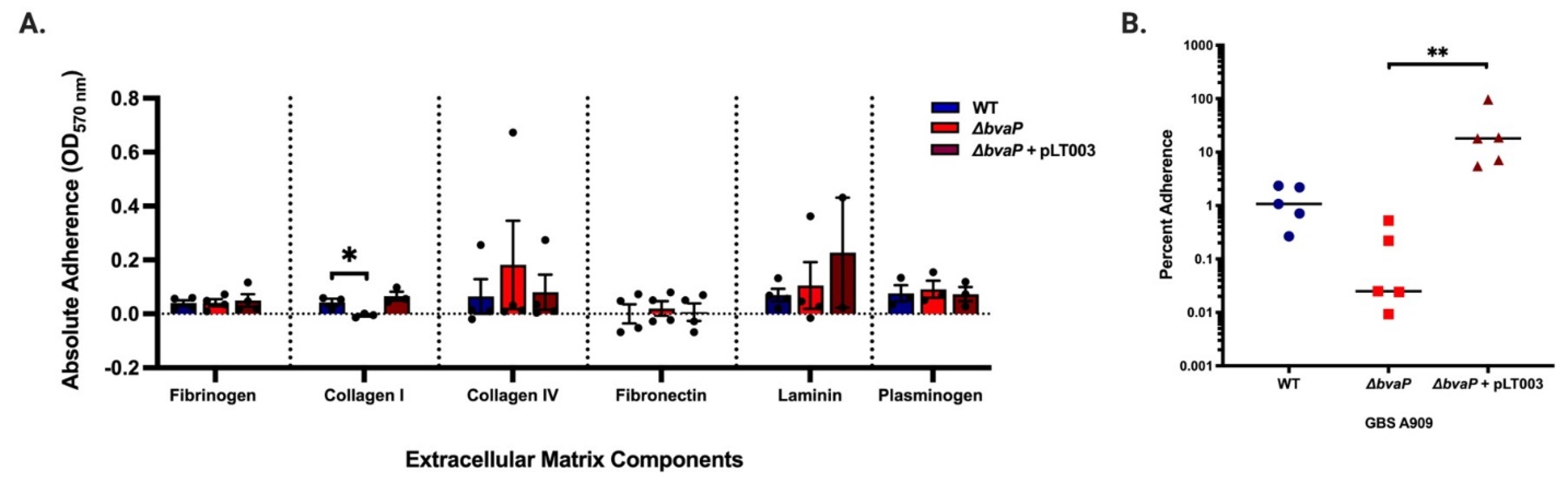

Supplement: FIG S4 [file msphere.00421-22-s0004.tif]

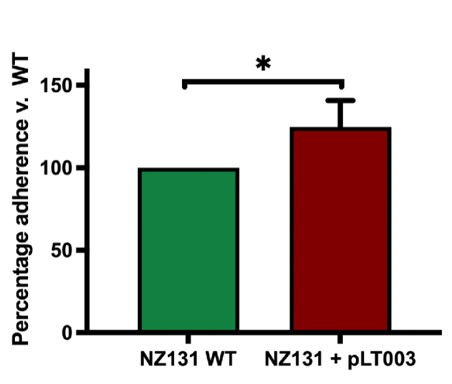

Supplement: FIG S5 [file msphere.00421-22-s0005.tif]

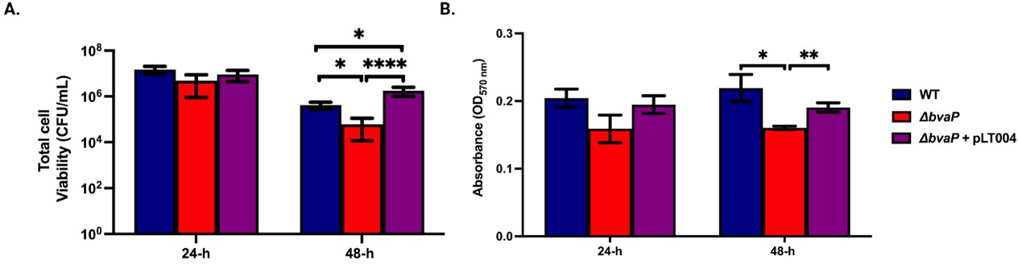

Supplement: FIG S6 [file msphere.00421-22-s0006.tif]
