# Supplementary material for: A Novel Conserved Protein in Streptococcus agalactiae, BvaP, Is Important for Vaginal Colonization and Biofilm Formation
Source: mSphere. 2022 Oct 11;7(6):e00421-22. doi: 10.1128/msphere.00421-22 (PMC9769775; doi:10.1128/msphere.00421-22)
Supplement: TABLE S1 [file msphere.00421-22-s0007.docx]

**Supplemental Table 1: Strains and Plasmids**

| **Strain Name** | **Description** | **Citation** |
| --- | --- | --- |
| A909 | Wild-type *S. agalactiae* type Ia/C clinical isolate | (1, 2) |
| A909Δ*bvaP* | A909 Δ*bvaP*:*specR* | This study |
| *E. coli* DH5α | Chemically competent cloning strain | NEB C2987H |
| NZ131 | Wild-type M49 *S. pyogenes* | (3) |
|  | | |
| **Plasmid Name** | **Description** | **Citation** |
| pMB*sacB* | pHY304 with p23-*sacB* cassette cloned into AvaII site, for sucrose-counter-selectable GBS mutagenesis | (4) |
| pLZ12Spec | Shuttle vector, Spec resistance | (5) |
| pJC303 | pLZ12Spec-based vector with *recA* constitutive promoter upstream of multiple cloning site | (6) |
| pLT001 | pMB*sacB*: *ΔbvaP* mutagenesis cassette | This study |
| pLT003 | Constitutive expression strain pJC303 + *bvaP* inserted after P_recA_ | This study |
| pLT004 | Complement pLZ12Spec + *bvaP* with 252 bp upstream containing  its putative native promoter | This study |

**References**

1. Lancefield RC, McCarty M, Everly WN. 1975. Multiple mouse-protective antibodies directed against group B streptococci. J Exp Med 142:165-79.

2. Tettelin H, Masignani V, Cieslewicz MJ, Donati C, Medini D, Ward NL, Angiuoli SV, Crabtree J, Jones AL, Durkin AS, Deboy RT, Davidsen TM, Mora M, Scarselli M, Margarit y Ros I, Peterson JD, Hauser CR, Sundaram JP, Nelson WC, Madupu R, Brinkac LM, Dodson RJ, Rosovitz MJ, Sullivan SA, Daugherty SC, Haft DH, Selengut J, Gwinn ML, Zhou L, Zafar N, Khouri H, Radune D, Dimitrov G, Watkins K, O'Connor KJ, Smith S, Utterback TR, White O, Rubens CE, Grandi G, Madoff LC, Kasper DL, Telford JL, Wessels MR, Rappuoli R, Fraser CM. 2005. Genome analysis of multiple pathogenic isolates of *Streptococcus agalactiae*: implications for the microbial "pan-genome". Proc Natl Acad Sci U S A 102:13950-5.

3. Simon D, Ferretti JJ. 1991. Electrotransformation of *Streptococcus pyogenes* with plasmid and linear DNA. FEMS Microbiol Lett 66:219-24.

4. Hooven TA, Bonakdar M, Chamby AB, Ratner AJ. 2019. A Counterselectable Sucrose Sensitivity Marker Permits Efficient and Flexible Mutagenesis in Streptococcus agalactiae. Appl Environ Microbiol 85.

5. Husmann LK, Scott JR, Lindahl G, Stenberg L. 1995. Expression of the Arp protein, a member of the M protein family, is not sufficient to inhibit phagocytosis of Streptococcus pyogenes. Infect Immun 63:345-8.

6. Chang JC, Federle MJ. 2016. PptAB Exports Rgg Quorum-Sensing Peptides in Streptococcus. PLoS One 11:e0168461.
