# Supplementary material for: A Novel Conserved Protein in Streptococcus agalactiae, BvaP, Is Important for Vaginal Colonization and Biofilm Formation
Source: mSphere. 2022 Oct 11;7(6):e00421-22. doi: 10.1128/msphere.00421-22 (PMC9769775; doi:10.1128/msphere.00421-22)
Supplement: TABLE S2 [file msphere.00421-22-s0008.docx]

**Supplemental Table 2: Primers**

| **PRIMER** | **SEQUENCE (5′ TO 3′)** |
| --- | --- |
| JC016_F | TTTCCCAGTCACGACGTTG |
| JC180_R | AGGCTATTGGTGTTTATGGC |
| LC060_F | CAATGGATGGGGATGGTG |
| LC061_R | CGCTGGTAAAACAAGAGGTTC |
| LC206_R | GAATTACCTCTTTCTTTTGGGT |
| LC208_F | TTTAATTGTGATGATCCTGCTCC |
| LC244_F | GGCCCCCCCTCGAGGTCGACGGTATACCTGCTTTTTTCCCTGATTC |
| LC246_F | AGAGGTAATTCTTATGTTTGGATCAGGAGTTG |
| LC247_R | CTACTTTATCTATTAATTTTTTTAATCTGTTATTTAAATAGTTTATAG |
| LC248_F | ATTAAAAAAATTAATAGATAAAGTAGATTAATGATTAAATAGTTAAG |
| LC249_R | GGCCGCTCTAGAACTAGTGGATCCCATTATTCGCCAACATTAGG |
| LC270_R | CACTTTGAAAAGTGTAACACTAGCT |
| LC273_F | GTAAGAAACGGTATCTGATTGATG |
| LC274_R | GCAAACAGCAAGAGTCCACCTC |
| LC280_R | TGTGGAATTGTGAGCGGATA |
| LC281_F | CCACTCTCAACTCCTGATCCA |
| LC282_R | GCAGTGGCTGAATCTTCTCC |
| LT001_R | CCAATTCGCCCTATAGTG |
| LT002_F | GTACCGGGCCCCCCCTCG |
| LT003_F | CTCACTATAGGGCGAATTGGACCTGCTTTTTTCCCTGATT |
| LT004_R | TCGAGGGGGGGCCCGGTACATTATTCGCCAACATTAGGTT |
| LT005_F | AAATGTTTAAATTAGGATCCAGATCTTCCTTCAGGTTATG |
| LT006_R | GTTTTTTCATGCGGCCGCCCTCCTAAAT |
| LT007_F | GGGCGGCCGCATGAAAAAACTTATTACAGAAAAAAAAG |
| LT008_R | GGATCCTAATTTAAACATTTAAAGTTGAATGCTG |
| LT009_R | ATAAGTTTTTTCATGCGGCCGCCCCTTACCAAA |
| LT011_F | TCTCTTGGTCGTCAGACTGA |
| LT012_R | AGGACCAGACATTACGAACTGGCACAGATG |
| LT013_F | TACCCATACGACGTCCCAGACTACGCTTAGATTAGGATCCAGATCTTCCTTCAGG |
| LT014_R | CTAAGCGTAGTCTGGGACGTCGTATGGGTAAACATTTAAAGTTGAATGCTGGTTA |
| LT016_F | GTTTTTTCATAAGAATTACCTCTTTCTTTTGG |
| LT024_F | GTCTCAACTGCTACTATTTGAAACTC |
| LT025_R | AGCTGAAACACCTTGTGTAGAA |
| LT116_F | ACTGGCTTTTATAAAAAAATTGAAAAAATGGTGG |
| LT118_F | AATAGGTACTAATCAAAATAATGAACTTTAATAAAATTGATTTAGACAATTG |
| LT119_R | CACCATTTTTTCAATTTTTTTATAAAAGCCAGTCATTAGGCC |
| LT126_R | TAAAGTTCATTATTTTGATTAGTACCTATTTTATATCC |
