# Supplementary material for: A Novel Conserved Protein in Streptococcus agalactiae, BvaP, Is Important for Vaginal Colonization and Biofilm Formation
Source: mSphere. 2022 Oct 11;7(6):e00421-22. doi: 10.1128/msphere.00421-22 (PMC9769775; doi:10.1128/msphere.00421-22)
Supplement: TEXT S1 [file msphere.00421-22-s0009.docx]

**SUPPLEMENTAL METHODS**

**RNA isolation.** Following overnight (o/n) growth in THY broth supplemented with the appropriate antibiotics, cultures were diluted 1:20 in fresh THY. Once the cells reached an optical density at 600 nm (OD_600_) of 0.4-0.7, 10 mL of cultures were spun down at the same OD, resuspended in 1 mL of RNALater (AM7024, Invitrogen), and incubated at room temperature for 5-10 minutes. Following RNALater treatment, cells were pelleted, and stored at -80^o^C until RNA extraction. RNA was extracted from bacterial pellets using the Ambion RiboPure Bacteria kit (AM1925; Thermo Fisher) according to the manufacturer’s protocol using 7 min of bead beating with a Bead-ruptor 12 (OMNI International) to lyse cells. RNA was DNase I treated using the same kit.

**Preparation of cDNA and qRT-PCR.** cDNA preparation was done using the iScript cDNA Synthesis Kit (1708890; BioRad) according to the manufacturer's instructions, including treatment with RNase H. cDNA was diluted between 1:2 and 1:5, depending on the concentration, and used for qPCR. All primers used in qPCR are listed in Table S2. qRT-PCR was done using the SYBR Green SSo for difficult templates (1725271, Biorad) and a CFX Connect Real Time PCR detection system (788BR01742, Bio-Rad). Gene-specific primers LC060/LC061 (*gyrA*), LT024/LT025 (*bvaP*) were used for amplification. *gyrA*, a housekeeping gene not seen to have differential expression during growth in the vaginal tract, was used as a reference gene. All samples were run in triplicate technical replicates on a single plate, and triplicate biological replicates were used to determine final statistics. Expression of *bvaP* was computed via the Pfaffl method using gDNA standard curves.

**Hydrophobicity assay.** Bacterial hydrophobicity was measured as previously described(1). Two mL of o/n cultures were pelleted by centrifugation at 8,000 x g for 2 min. Pellets were washed twice with PBS then resuspended in 2 mL PBS. 500 μL of o-xylene (Fisher Scientific, AAA11358AP) was added to each sample. Each sample had a negative control consisting only of 2 mL bacteria suspension. Samples were capped and vortexed vigorously for 15 sec. The layers were allowed to separate for 10 min. Hydrophobicity was evaluated through the measurement of the absorbance at OD_600_ of the aqueous fraction divided by its respective control multiplied by 100 then subtracted from 100. The assay was performed in biological triplicate.

**Hemolysis.** O/n cultures of the WT, mutant and complemented strains were streaked onto a blood agar plate followed by incubation o/n at 37°C. Hemolytic activity was observed by the lysis of blood cells in the medium indicated by a zone of clearing where colonies have grown.

**Capsule Stain.** Broth cultures were grown o/n and 10 μL was added to a cleaned microscope slide. The broth was diluted 1:1 with sterile distilled water then smeared and allowed to air dry. GBS capsule was stained using the Anthony direct-dry staining method, previously described (2). A 1% crystal violet solution (w/v) was used to flood the prepared slides for 2 min. Slides were gently rinsed with 20% copper (II) sulfate solution (w/v) then allowed to air dry and viewed with oil immersion below and above the coverslip at 1,000x magnification on an Olympus U-LHLEDC.

**Biofilm Quantification.** Biofilms were grown as described in the methods. Following growth, GBS cells were removed from coverslips by incubating with 500 μL of 0.05% Trypsin-EDTA and 0.25% Triton-X100 for 5 min at 37°C. Bacterial counts in each sample were determined by dilution plating on THY agar plates. Quantification was performed in technical triplicate on a single plate and in biological triplicate. For the crystal violet biofilm assay, 1 mL of 1% crystal violet (CV) solution (w/v) was added to each well containing washed biofilm on coverslips and incubated for 15 min on the bench. Following the removal of the CV from the wells, coverslips were washed with PBS three times then incubated with 95% ethanol for 5 min. Absorbance was measured at 570 nm on Tecan Infinite PRO spectrophotometer microplate reader.

**Immunofluorescence microscopy.** Log phase bacterial cells were centrifuged, washed, and suspended in PBS. After incubation for 30 min at room temperature with 1:1000 dilution of anti-HA.11 (BioLegend, 901501) cells were centrifuged again and resuspended in PBS. Cells were incubated for another 30 minutes at room temperature in the dark with 1:100 goat anti-mouse FITC-conjugated antibody (Jackson ImmunoResearch Laboratories Inc, 115-095-003). Bacterial cells were washed and incubated for 5-10 minutes with wheat germ agglutinin CF 633 conjugate (WGA; Biotium, 29024-1 and 4′,6-diamidino-2-phenylindole (DAPI; Fisher, 5.08741.0001) at a working concentration of 2 μg/ mL. Bacteria were observed under an Olympus U-LHLEDC epifluorescent microscope equipped with an L1-5 filter and viewed under an Olympus DP74 UPlan FLN 100×/1.30 oil objective.

**References**

1. Campeau A, Uchiyama S, Sanchez C, Sauceda C, Nizet V, Gonzalez DJ. 2021. The S Protein of Group B Streptococcus Is a Critical Virulence Determinant That Impacts the Cell Surface Virulome. Front Microbiol 12:729308.

2. Breakwell DP, Moyes RB, Reynolds J. 2009. Differential staining of bacteria: capsule stain. Curr Protoc Microbiol Appendix 3.
